# Effect of abacavir on sustained virologic response to HCV treatment in HIV/HCV co-infected patients, Cohere in Eurocoord

**DOI:** 10.1186/s12879-015-1224-1

**Published:** 2015-11-04

**Authors:** For the Hepatitis C- working group for COHERE in Eurocoord, Colette Smit, Joop Arends, Lars Peters, Antonella d’Arminio Montforte, Francois Dabis, Robert Zangerle, George Daikos, Christina Mussini, Josep Mallolas, Stephane de Wit, Annelies Zinkernagel, Jaime Cosin, Genevieve Chene, Dorthe Raben, Jürgen Rockstroh

**Affiliations:** Stichting HIV Monitoring, Meibergdreef 9, 1105AZ Amsterdam, The Netherlands

**Keywords:** HCV/HIV co-infection, HCV treatment, HCV treatment response, Abacavir

## Abstract

**Background:**

Contradicting results on the effect of abacavir (ABC) on hepatitis C virus (HCV) treatment responses in HIV/HCV co-infected patients have been reported. We evaluated the influence of ABC on the response to pegylated interferon (pegIFN) and ribavirin (RBV)-containing HCV treatment in HIV/HCV co-infected patients in a large European cohort collaboration, including data from different European countries.

**Methods:**

HIV/HCV co-infected patients were included if they were aged ≥16 years, received pegIFN alfa-2a or 2b and RBV combination treatment and were enrolled in the COHERE cohort collaboration. Logistic regression was used to evaluate the impact of abacavir on achieving a sustained virologic response (SVR) to HCV treatment.

**Results:**

In total 1309 HIV/HCV co-infected patients who had received HCV therapy were included, of whom 490 (37 %) had achieved an SVR. No statistically significant difference was seen for patients using ABC-containing regimens compared to patients using an emtricitabine + tenofovir (FTC + TDF)-containing backbone, which was the most frequently used backbone. In the multivariate analyses, patients using a protease inhibitor (PI)-boosted regimen were less likely to achieve an SVR compared to patients using a non-nucleoside reverse-transcriptase inhibitor (NNRTI)-based regimen (OR: 0.61, 95 % CI: 0.41–0.91). The backbone combinations zidovudine&lamivudine (AZT + 3TC) and stavudine&lamivudine (d4t + 3TC) were associated with lower SRV rates (0.45 (0.24–0.82) and 0.46 (0.22–0.96), respectively).

**Conclusion:**

The results of this large European cohort study validate that SVR rates are generally not affected by ABC. Use of d4T or AZT as part of the HIV treatment regimen was associated with a lower likelihood of achieving an SVR.

**Electronic supplementary material:**

The online version of this article (doi:10.1186/s12879-015-1224-1) contains supplementary material, which is available to authorized users.

## Background

Until recently, treatment for hepatitis C (HCV) consisted of a combination of pegylated interferon (pegIFN) and ribavirin (RBV), combined more recently with boceprevir and telaprevir or with some of the new direct-acting antivirals (DAA)-containing regimens. In HIV/HCV co-infected patients, treatment for HCV is often administrated concomitantly with combination antiretroviral therapy (cART).

Earlier studies have reported contradicting results regarding the effect of an abacavir-based cART regimen (ABC) on HCV treatment response. For example, some studies found ABC has a negative effect on sustained virologic response (SVR) in the presence of HCV therapy [1–3]. This may be because RBV and ABC share intracellular pathways [4], which could, theoretically, affect RBV drug concentrations and therefore the effectiveness of RBV. However, other studies found no difference in SVR between patients who received an ABC-containing regimen in combination with HCV treatment and those who did not use ABC concomitantly with HCV treatment [5–8]. These discrepancies might be due to the relatively small samples sizes used in the above-mentioned studies. One larger study, conducted by Berenguer et al. has already been carried out and found that ABC was not associated with a lower response to HCV treatment [9].

This issue of contradicting results regarding the interaction between ABC and RBV remains important for two reasons. First, even in interferon-free regimens, RBV will often be used with a large number of new DAAs. Furthermore, following the introduction of the HIV integrase inhibitor dolutegravir, which is co-formulated with ABC/3TC in a fixed-dose combination, use of ABC with 3TC is expected to increase. Therefore, to validate the results of the earlier large cohort study by Berenguer et al. we aimed to examine the influence of ABC on the response to pegIFN and RBV-containing HCV treatment in HIV/HCV co-infected patients in a large European cohort collaboration comprising data from different European countries.

## Methods

### Study population

Individuals included in this study were enrolled in HIV cohorts participating in the Collaboration of Observational HIV Epidemiological Research in Europe (COHERE). COHERE is a collaboration of 33 cohorts across Europe and is part of the EuroCoord network (www.cohere.org and www.EuroCoord.net). The aim of COHERE is to conduct epidemiological research into the prognosis and outcome of HIV-infected individuals, which the individual participating cohorts cannot address themselves because of small sample sizes. Participating cohorts were approved by a local ethics committee or institutional review board. The study included all HIV-positive individuals with a positive HCV RNA test result who were aged 16 years or older at the time of HIV diagnosis and who had started cART after 1 January 1998. Twelve cohorts across 9 European countries, totalling 1309 patients, provided data for the present analysis: AHIVCOS (*n* = 39), AMACS (*n* = 9), ATHENA (*n* = 140), BONN/COLOGNE (*n* = 3), EUROSIDA (*n* = 219), HEPAVIH (*n* = 287), ICONA (*n* = 102), MODENA (*n* = 37), PISCIS (*n* = 49), The Swiss Cohort Study (*n* = 285), St Pierre Cohort Brussels (*n* = 25), VACH (*n* = 114). Participating cohorts adhere to the local ethics requirements, cohorts with ethics approval and individual patient written informed consent are AHIVCOS, AMACS, HEPAVIH, ICONA, MODENA, The Swiss Cohort Study, Eurosida and PISCIS. The remaining cohorts did not require ethics approval according to the national legislation (Further details of ethical requirements can be found in Additional file 1). All patients included in the present analysis received anti-HCV treatment that included the combined use of pegIFN alfa-2a or 2b and RBV at standard doses, with a usual duration of 24 or 48 weeks, depending on HCV genotype. Data on the use of boceprevir and telaprevir was not available at the time of database closure. As treatment response in patients with an acute HCV infection might differ from the treatment response in chronically infected patients, patients with less than 6 months between the first available positive HCV test result and the start of anti-HCV treatment (i.e., acute HCV infection) were excluded (*n* = 183). To assess the extent of liver fibrosis, aspartate amino transferase-to-platelet ratio (APRI) scores were calculated. APRI is a non-invasive method to assess liver fibrosis that combines AST levels and platelets counts [10]; an APRI score >1.5 correlates with severe fibrosis. The main outcome of interest was SVR, defined as a negative HCV RNA test result 24 weeks after treatment discontinuation in patients treated for chronic HCV infection.

### Statistical analysis

Logistic regression was used to identify factors associated with achieving SVR. Treatment of HIV was categorised as protease inhibitor (PI)-unboosted, PI-boosted, non-nucleoside reverse transcriptase inhibitor (NNRTI)-based, PI/NNRTI-based regimens and a fifth category including patients who had started cART after starting HCV treatment. The NRTI backbone was categorised according to the most commonly-used combinations: abacavir + lamivudine (ABC + 3TC), zidovudine + lamivudine (AZT + 3TC), emtricitabine + tenofovir (FTC + TDF), tenofovir + lamivudine (TDF + 3TC), tenofovir + abacavir (TDF + ABC), stavudine + lamivudine (d4T + 3TC), other combinations, and, finally, a separate group of 242 patients who started cART after receiving HCV treatment. To account for the shift from fixed dosing of RBV to weight-based use of RBV, we assumed that most patients received weight-based RBV doses from 2006 onwards and, therefore, analyses were adjusted for calendar year of starting HCV treatment.

Baseline characteristics of patients achieving an SVR were compared to non-responders using Student’s *t*-test for the continuous variables and the Chi-square test for the categorical variables. Predictors for achieving an SVR were assessed by calculating odds ratios (OR) with a 95 % confidence interval (CI) using a logistic regression model. Multivariate models were built using forward-stepwise techniques. Variables with a *p*-value <0.2 in the univariate analyses were considered as potential independent determinants and included in the multivariate analysis. A *p*-value <0.05 was considered statistically significant. Interactions in the final model were tested. All analyses were performed using SAS version 9.3.

## Results

### Study population

We analysed data from 1309 HIV-infected patients with a chronic HCV infection who had been prescribed a combination of pegIFN and RBV between 1998 and 2011. The most common genotype was HCV genotype 1 (*n* = 536, 40 %) (Table 1). The median duration of anti-HCV treatment was 31 weeks (inter quartile range (IQR): 16–49). Of the 1309 patients, 868 completed the full course of therapy (i.e., 24–48 weeks) with pegIFN and RBV, whereas 441 prematurely discontinued treatment before week 24 because of side effects or lack of virologic response. The proportion of patients who discontinued HCV treatment before week 24 was higher among patients on PI and PI-boosted regimens (42 and 38 %, respectively) compared to patients on an NNRTI-containing regimen (30 %, *p* = 0.09). The proportion of patients who prematurely discontinued HCV treatment did not differ between the different NRTI-backbone combinations (*p* = 0.23). Haemoglobin levels were more likely to drop by more than 2.5 g/dl compared to baseline in patients who prematurely discontinued HCV treatment (46 %) than in those who received HCV treatment for 24 or 48 weeks (31 and 23 %, respectively; *p* < 0.0001).

**Table 1:** Demographic and clinical characteristics at start of anti-hepatitis C virus (HCV) treatment of chronically HIV/HCV co-infected patients, 1998-2011

	Sustained virologic response			
	Total	No	Yes	*p*-value
Number of patients (%)	1309	819 (63)^a^	490 (37)^a^	
Age baseline, years	42 (38–46)	42 (38–46)	42 (37–46)	0.04
Baseline BMI				<0.0001
< 25	329 (25^b^)	234 (71)	95 (29)	
> =25	123 (9)	96 (78)	27 (22)	
Unknown	857 (66)	489 (57)	368 (43)	
Gender				0.57
Male	960 (73)	605 (63)	355 (37)	
Female	349 (39)	214 (61)	135 (39)	
HIV transmission route:				0.05
Men who have sex with men	175 (13)	91 (52)	84 (48)	
Male, injection drug use (IDU)	595 (45)	393 (66)	202 (34)	
Female IDU	209 (16)	127 (61)	82 (39)	
Male heterosexual	84 (6)	55 (65)	29 (35)	
Female heterosexual	96 (7)	62 (65)	34 (35)	
Male other/unknown	106 (8)	66 (62)	40 (38)	
Female other/unknown	44 (3)	25 (57)	19 (43)	
Region of origin:				0.06
Western	1101 (84)	700 (64)	401 (36)	
Other	116 (9)	72 (62)	44 (38)	
Unknown	92 (7)	47 (51)	45 (49)	
CD4 at baseline (cells/μl)				
0–349	264 (20)	163 (62)	101 (38)	0.02
350–499	306 (23)	194 (63)	112 (37)	
> =500	491 (38)	288 (59)	203 (41)	
Missing	248 (19)	174 (70)	74 (30)	
Nadir CD4 (cells/ μl)				<0.001
< 200	689 (53)	434 (63)	255 (37)	
> =200	583 (45)	381 (65)	202 (35)	
Missing	37 (3)	4 (11)	33 (89)	
HIV RNA levels at baseline (copies/ml)				0.99
< =400	1082 (83)	677 (63)	405 (37)	
> 400	227 (17)	142 (63)	85 (37)	
Hepatitis B virus co-infection				0.64
No	1214 (93)	762 (63)	452 (37)	
Yes	53 (4)	30 (57)	23 (43)	
Unknown	42 (3)	27 (64)	14 (33)	
HCV RNA load at baseline				<0.0001
< 600,000	222 (17)	99 (45)	123 (55)	
≥ 600,000	724 (55)	485 (67)	239 (33)	
Missing	363 (28)	235 (65)	128 (35)	
HCV genotypes				<0.0001
1	82 (6)	53 (65)	29 (35)	
1a	307 (23)	234 (76)	83 (27)	
1b	147 (11)	108 (73)	39 (27)	
2&3	315 (24)	154 (49)	161 (51)	
4	143 (11)	103 (72)	40 (28)	
Other/unknown	315 (24)	177 (56)	138 (44)	
APRI^c^ score:				<0.0001
< 0.5	409 (31)	206 (50)	203 (50)	
0.5–1.5	426 (32)	312 (73)	114 (27)	
> =1.5	195 (15)	155 (79)	40 (21)	
Unknown	279 (21)	146 (52)	133 (48)	
Decline in haemoglobin (g/dl)				0.0017
No decline	179 (14)	126 (70)	53 (30)	
< =2.5	227 (17)	157 (69)	70 (31)	
> 2.5	230 (18)	127 (55)	103 (45)	
Missing	637 (49)	409 (64)	264 (41)	
Duration of HCV treatment in weeks				<0.0001
< =24	496 (38)	390 (79)	106 (21)	
24-48	437 (33)	242 (55)	195 (45)	
> 48	376 (29)	187 (50)	189 (50)	
Calendar year of start HCV treatment				<0.0001
< =2003	266 (20)	201 (76)	65(24)	
2003–2006	421 (32)	258 (61)	163 (39)	
> =2006	622 (48)	360 (58)	262 (42)	
cART regimen				0.003
PI	158 (12)	104 (66)	54 (34)	
Boosted PI	444 (34)	296 (67)	148 (33)	
NNRTI	284 (22)	154 (54)	130 (46)	
PI + NNRTI	50 (6)	35 (70)	15 (30)	
No PI and/or NNRTI	131 (10)	90 (69)	41 (31)	
no cART	242 (18)	140 (58)	102 (42)	
Backbone				<0.0001
Start after HCV treatment	242 (18)	140 (58)	102 (42)	
ABC + 3TC	189 (14)	117 (62)	72 (38)	
AZT + 3TC	140 (11)	99 (62)	41 (29)	
FTC + TDF	262 (20)	147 (56)	115 (44)	
TDF + 3TC	130 (10)	68 (52)	62 (48)	
TDF + ABC	44 (3)	27 (61)	17 (39)	
d4T + 3TC	90 (7)	68 (76)	22 (24)	
other	212 (16)	153 (72)	59 (28)	

^a^percentage in these colums are representing the percentage of the total number of patients in a specific category/row

^b^Percentage from total number of patients included in this study (*n* = 1309)

^c^APRI, aspartate amino transferase-to-platelet ratio; PI, protease inhibitor; NNRTI, non-nucleoside reverse transcriptase; NRTI, nucleoside analog reverse-transcriptaseinhibitor; ABC, abacavir, 3TC, lamivudine; AZT, zidovudine; FTC, emtricitabine; TDF, tenofovir; d4T, stavudine

Baseline clinical characteristics at the start of HCV treatment of the included patients are shown in Table 1. Thirty-four percent of the patients used a PI-boosted cART regimen. A combination of FTC + TDF was the most frequently used NRTI backbone (20 %). In total, 233 patients used an ABC-containing NRT combination: 189 used ABC + 3TC (14 %) and 44 used ABC + TDF (3 %).

### SVR

In total, 490 (37 %) patients achieved SVR. The proportion of patients with a baseline CD4 cell count ≥500 cells/μl was significantly higher among patients who achieved an SVR (41 % vs 35 %; *p* = 0.02). Patients with genotype 1 or 4 were significantly less likely to achieve SVR than patients with genotypes 2 or 3 (*p* < 0.0001).

The SVR rates ranged from 30 % for patients on a regimen that included a PI and an NNRTI to 46 % for those who were treated with an NNRTI-containing combination (*p* = 0.03). When stratified according to NRTI-backbone, the SVR rate was 24 % amongst patients using a d4T&3TC backbone, and the SVR rate was 48 % in patients using a TDF&3TC backbone. The SVR rate was 38 % for patients with a ABC&AZT backbone and 39 % for those with a TDF&ABC backbone.

### Predictors of SVR

Table 2 shows the predictive factors for SVR in HCV/HIV co-infected patients receiving anti-HCV treatment. In the univariate analyses, ABC-containing backbones were not associated with a higher or lower likelihood of achieving an SVR. Longer duration of HCV treatment compared to less than 24 weeks of treatment, men who have sex with men, HCV genotypes 2 and 3, lower APRI score, and a larger decline in haemoglobin levels were associated with a higher likelihood of achieving an SVR. Starting HCV treatment before 2003 was significantly associated with a lower likelihood of achieving an SVR compared to starting in or after 2006. Patients who started between 2003 and 2005 were non-significantly less likely to achieve an SVR. Patients using a PI-boosted cART regimen or an AZT + 3TC NRTI backbone were also less likely to achieve an SVR.

**Table 2:** Association of predictive factors with sustained virologic response among patients receiving anti-hepatitis C virus (HCV) treatment in chronically HCV/HIV co-infected patients, COHERE collaboration, 1998–2011, using logistic regression with forward selection of the variables included in the multivariate model

		Univariate	*p*-value	Multivariate	*p*-value
		Odds ratio (95 % confidence interval)		Odds ratio (95 % confidence interval)	
Age at start HCV treatment (years)	16–34	1		1	
	35–49	0.71 (0.51–0.99)	0.04	0.80 (0.52–1.23)	0.29
	> = 50	0.68 (0.43–1.07)	0.10	0.58 (0.32–1.04)	0.06
Duration HCV treatment in weeks	<24	1		1	
	24–48	2.97 (2.23–3.95)	<0.0001	2.85 (2.0–4.05)	<0.0001
	>48	3.72 (2.77–4.99)	<0.0001	4.92 (3.43–7.08)	<0.0001
Calendar year of starting HCV treatment	<=2003	0.45 (0.32–0.64)	<0.0001	0.41 (0.29–0.71)	0.0008
	2003–2005	0.90 (0.69–1.18)	0.27	1.10 (0.78–1.59)	0.62
	> = 2006	1		1	
BMI	<25	1		1	
	> = 25	0.69 (0.42–1.13)	0.14	1.01 (0.58–1.76)	0.97
	unknown	1.85 (1.41–2.44)	<0.0001	6.12 (3.99–9.38)	<0.0001
Gender	Male	1	0.57		-
	Female	1.08 (0.84–1.38)			
HIV transmission route:	MSM	1.80 (1.28–2.53)	0.0008		-
	Male injection drug us (IDU)	1			
	Female IDU	1.26 (0.91–1.74)	0.17		
	Male heterosexual	1.03 (0.63–1.66)	0.92		
	Female heterosexual	1.07 (0.68–1.68)	0.77		
	Male other/unknown	1.18 (0.77–1.81)	0.45		
	Female other/unknown	1.48 (0.80–2.75)	0.21		
Region of origin:	Western	1			-
	Other	1.07 (0.72–1.58)	0.75		
	Unknown	1.67 (1.09–2.56)	0.02		
HIV RNA levels at baseline (copies/ml)	<=400	1.00 (0.74–1.34)	0.99		-
	>400	1			
Hepatitis B virus co-infection	No	1			-
	Yes	1.29 (0.74–2.25)	0.37		
	unknown	0.94 (0.49–1.78)	0.58		
Nadir CD4 (cells/μl)	<200	1		1	
	> = 200	0.90 (0.72–1.14)	0.38	0.80 (0.57–1.12)	0.19
CD4 at baseline (cells/μl)	0–349	1		1	
	350–499	0.93 (0.66–1.31)	0.68	0.69 (0.45–1.07)	0.09
	> = 500	1.14 (0.84–1.54)	0.40	0.94 (0.62–1.42)	0.75
	missing	0.69 (0.48–0.99)	0.04	0.10 (0.05–0.19)	<0.0001
HCV RNA load at baseline	<600,000	2.52 (1.86–3.43)	<0.0001	2.06 (1.38–3.06)	0.0004
	≥600,000	1		1	
	missing	1.11 (0.85–1.44)	0.46	1.41 (0.90–2.20)	0.13
HCV genotypes	1	0.52 (0.32–0.87)	0.02	0.27 (0.14–0.51)	<0.0001
	1a	0.35 (0.25–0.50)	<0.0001	0.25 (0.17–0.39)	<0.0001
	1b	0.35 (0.23–0.53)	<0.0001	0.28 (0.16–0.47)	<0.0001
	2 & 3	1		1	
	4	0.37 (0.24–0.60)	<0.0001	0.32 (0.19–0.54)	<0.0001
	Other/unknown	0.74 (0.55–1.02)	0.69	0.70 (0.46–1.05)	0.023
APRI^a^ score	<0.5	1		1	
	0.5–1.5	0.37 (0.28–0.50)	<0.0001	0.41 (0.29–0.58)	<0.0001
	> = 1.5	0.26 (0.18–0.39)	<0.0001	0.24 (0.15–0.39)	<0.0001
	unknown	0.92 (0.68–1.25)	0.61	0.61 (0.40–0.94)	0.023
Decline in haemoglobin (g/dl)	No decline	1		1	
	<=2.5	1.06 (0.69–1.62)	0.78	0.89 (0.52–1.51)	0.66
	>2.5	1.93 (1.28–2.92)	0.002	1.36 (0.81–2.28)	0.24
	missing	1.53 (1.08–2.19)	0.02	4.66 (2.81–7.72)	<0.0001
cART use:	PI	0.62 (0.41–0.92)	0.02	1.04 (0.62–1.75)	0.87
	Boosted PI	0.59 (0.44–0.80)	0.0008	0.61 (0.41–0.91)	0.02
	NNRT	1		1	
	PI + NNRT	0.51 (0.27–0.97)	0.04	0.65 (0.29–1.44)	0.29
	No PI and/or NNRT	0.54 (0.39–0.84)	0.006	0.78 (0.44–1.39)	0.39
	Start after HCV treatment	0.86 (0.61–1.22)	0.40	NA^b^	
NRTI backbone^a^	Start after HCV treatment	0.93 (0.65–1.33)	0.69	0.96 (0.57–1.61)	0.34
	ABC + 3TC	0.79 (0.54–1.15)	0.22	0.74 (0.45–1.24)	0.25
	AZT + 3TC	0.53 (0.34–0.82)	0.004	0.44 (0.24–0.80)	0.007
	FTC + TDF	1		1	
	TDF + 3TC	1.17 (0.76–1.78)	0.47	0.76 (0.44–1.32)	0.33
	TDF + ABC	0.81 (0.42–1.55)	0.51	0.79 (0.35–1.78)	0.57
	d4T + 3TC	0.41 (0.24–0.71)	0.001	0.46 (0.22–0.96)	0.04
	other	0.50 (0.34–0.73)	0.0003	0.54 (0.32–0.91)	0.02

^a^APRI, aspartate amino transferase-to-platelet ratio; PI, protease inhibitor; NNRTI, non-nucleoside reverse transcriptase; NRTI, nucleoside analog reverse-transcriptaseinhibitor; ABC, abacavir, 3TC, lamivudine; AZT, zidovudine; FTC, emtricitabine; TDF, tenofovir; d4T, stavudine

^b^Odds ratio could not be calculated due to collinearity with the NRTI backbone category ‘start after HCV treatment’

In the multivariate analyses, after adjustment for differences in clinical and demographic variables, there remained no association between ABC-containing regimens and a higher or lower likelihood of achieving an SVR. A boosted PI regimen remained significantly associated with a lower probability of achieving an SVR compared to NNRTI-based regimens. Overall, the different backbones, and most importantly the use of ABC, were not associated with a low SVR after adjustment for differences in clinical and demographical variables, with the exception of the AZT + 3TC and d4t + 3TC combinations. Patients using these backbones were less likely to achieve an SVR (0.45 (0.24–0.82) and 0.46 (0.22–0.96), respectively). No statistically significant difference was seen between patients using ABC -containing regimens and those using an FTC + TDF-containing backbone. No statistically significant difference was observed between patients who started cART after receiving HCV treatment and those using an FTC + TDF-containing backbone. Finally, earlier calendar year of starting HCV treatment remained significantly associated with a lower odds of achieving an SVR.

## Discussion

The results of this study, conducted in an unselected cohort of HCV/HIV co-infected patients from different countries in Europe, showed no difference in response to HCV treatment in patients using an ABC-containing regimen compared to those using an FTC + TDF-containing backbone. Overall, 37 % of patients achieved an SVR, compared to 29 % of patients on a boosted PI regimen. The response to HCV treatment did not differ between patients who used cART and those who did not.

Earlier studies have shown contradicting results for the effect of ABC [2, 3, 6–8]. The results of our study, conducted in a large multi-cohort study, validate those of an earlier large cohort study into the effect of cART on HCV treatment outcome [9]. In our study, the concomitant use of ABC + 3TC or ABC + TDF and HCV treatment did not result in different SVR rates compared to the concomitant of TDF + FTC and HCV treatment, which was most frequently used in this patient population. In terms of the NRTIs used during HCV treatment, in this study AZT in combination with 3TC and d4T in combination with 3TC negatively affected the response to HCV treatment. This negative effect of these NRTIs might be due not only to the interference of d4T with RBV [11], but also the effect of AZT on lowering haemoglobin levels [12]. Moreover, according to the guidelines from the European AIDS Clinical Society, d4T and AZT use should be avoided during pegIFN and RBV treatment [13], as both PEG-IFN and RBV are also commonly known to decrease haemoglobin levels [14, 15]. In fact, anaemia is frequently observed in patients during HCV therapy [12] and is often a cause for RBV dose reductions or early discontinuation of HCV treatment, which negatively impacts SVR rates [16]. On the other hand, lower haemoglobin levels have been shown to be associated with higher SVR rates [17]. This could reflect adequate weight-based RBV dosing accompanied by more side effects such as declining haemoglobin levels. Although we had no data on RBV dosing, haemoglobin measurements were available for half the patients. Consequently, we were able to calculate changes in haemoglobin levels and to use the decline in haemoglobin levels as a proxy for changes in RBV doses, assuming that a stronger decline in haemoglobin levels might be a marker for higher RBV doses. As a result of including this haemoglobin change in our analyses we observed higher SVR rates in patients with a haemoglobin decline of 2.5 g/dl or more from baseline, which could reflect higher RBV levels. In addition, the results of a sensitivity analysis including patients with a haemoglobin decline of >2.5 g/dl showed that patients using the combination of AZT and 3TC remained less likely to achieve an SVR compared to patients who used FTC + TDF. Although this result was not statistically significant, the effect of AZT on lowering haemoglobin levels could have attributed to higher toxicity and early discontinuation.

Several reports have shown that weight-based dosing of RBV is more effective than flat dosing of RBV, and that adequate dosing of RBV is crucial to maximising HCV treatment response [18, 19]. Consequently, there has been a shift from flat dosing to weight-based dosing over time, with most patients receiving weight-based RBV from 2006 onwards. To account for this shift and address the lack of information on RBV dosing in our study, we included calendar time of starting HCV treatment.

The composition of cART regimens has changed over time. In recent years, there has been a drop in the use of AZT and d4T in cART regimens; in fact the European AIDS clinical society guidelines no longer recommend inclusion of AZT and d4T in initial regimens [13]. Moreover, with the introduction of the HIV integrase inhibitor dolutegravir, use of ABC in combination with 3TC is expected to increase in the future. Therefore, to evaluate the impact of these changes over calendar time we included an interaction term between calendar time and NRTI backbones in the logistic regression model. This analysis found no statistically significant interaction and therefore we assumed that the effect of cART regimens on HCV treatment response does not vary with calendar time.

Our study found a significantly lower SVR in patients on a boosted PI regimens compared to those on an NNRTI-based regimen. This confirms previous reports of an association between a PI-based regimen and lower SVR rates [20]. Furthermore, Berenguer et al. also found that patients on a boosted PI were less likely to achieve an SVR, although this result was statistically non-significant [9]. The significant difference between a boosted PI-based regimen and an NNRTI-based regimen in our study might be due to differences in patient characteristics in the two groups: patients on a boosted PI-based regimen had significant lower CD4 counts at the time of cART initiation than patients on an NNRTI cART regimen. Since boosted PI regimens are likely to be prescribed to patients who experienced virological failure on earlier cART regimens, these patients may have been infected with HIV for a longer period of time and may have had more advanced HIV disease progression. It is also likely that these patients had been chronically infected with HCV for a longer period of time. As a result, these patients may have had a higher degree of liver damage, as progression to liver disease is common with HCV and known to be accelerated in the presence of HIV [21, 22], and therefore might have been less likely to achieve an SVR [23]. To account for the progression to liver disease and for advanced HIV disease, in the final multivariate model we included not only the APRI score, which has been shown to be a reliable marker for predicting hepatic fibrosis in HIV/HCV co-infected patients [10], but also nadir CD4 cell count. After adjustment for differences in advanced HIV and liver disease, our study still shows a trend towards a lower probability of achieving an SVR in patients on a boosted PI regimen. Furthermore, although not statistically significant, the discontinuation rate of HCV treatment was somewhat higher in patients receiving a PI or PI-boosted cART regimen, compared to patients receiving an NNRTI cART regimen.

The primary aim of our study was to examine the influence of ABC on the response to pegIFN and RBV-containing HCV treatment in patients already receiving cART prior to HCV treatment. However, we also included a group of patients who started cART after receiving HCV treatment. When we compared this group to those who were already using an FTC + TDF-containing backbone prior to starting HCV treatment, we found no statistically significant difference in SVR response rates. An explanation for this finding could be that the group of patients who started cART after HCV treatment were relatively healthy and not yet in need for HIV treatment. This assumption is supported by the large number of these patients with a high CD4 cell count at the time of HCV treatment initiation: median CD4 cell count in this group was 494 cells/mm^3^ (interquartile range: 387–651).

Finally, the number of new DAAs is increasing and, consequently, the number of treatment options that rule out the need for pegIFN will also increase substantially. In view of this development, the interaction between pegIFN and ABC has become less relevant. Furthermore, as the result of the growing number of treatment options, including DAA combinations without RBV, it is also likely that fewer patients will be treated with a RBV- containing DAA combination in the future, which might limit the clinical importance of the present findings. Nonetheless, we believe knowledge regarding a possible interaction between RBV and ABC remains important as some of the new DAAs may still be used in combination with RBV. Moreover, the high costs of these new DAAs could limit access to these treatment options in some regions, which might result in RBV still being used in combination with ABC in certain settings.

## Conclusions

The results of this large European cohort study validate those of another large cohort study by showing that SVR rates are generally not affected by ABC. Use of d4T or AZT as part of the HIV treatment regimen was associated with a lower likelihood of achieving an SVR, which, in the case of AZT, might be related to its propensity to induce anaemia. A potential negative impact of a boosted PI regimen may warrant further evaluation. Finally, we found no evidence of a harmful effect of ABC-containing regimens in future DAA and RBV combinations.

## Additional file

Additional file 1:
**Ethics requirements for cohorts and studies for Cohere in EuroCoord.** (DOC 29 kb)

## Abbreviations

HCV: Hepatitis C virus
cART: combination antiretroviral therapy
Cohere: Collaboration of Observational HIV Epidemiological Research in Europe
SVR: Sustained virological response
pegIFN: pegylated interferon
RBV: Ribavirin
APRI: Aspartate amino transferase-to-platelet ratio
PI: Protease inhibitor
NNRTI: Non-nucleoside reverse transcriptase
NRTI: Nucleoside analog reverse-transcriptaseinhibitor
ABC: Abacavir
3TC: Lamivudine
AZT: Zidovudine
FTC: Emtricitabine
TDF: Tenofovir
d4T: Stavudine
OR: Odds ratio
CI: Confidence interval
DAA: Direct-acting antivirals.
