# Supplementary material for: Effect of abacavir on sustained virologic response to HCV treatment in HIV/HCV co-infected patients, Cohere in Eurocoord
Source: BMC Infect Dis. 2015 Nov 4;15:498. doi: 10.1186/s12879-015-1224-1 (PMC4634902; doi:10.1186/s12879-015-1224-1)
Supplement: Additional file 1: — Ethics requirements for cohorts and studies for Cohere in EuroCoord. (DOC 29 kb) [file 12879_2015_1224_MOESM1_ESM.doc]

**Ethics requirements for cohorts and studies for Cohere in EuroCoord**

Cohort participants are recruited to individual cohorts within Cohere through their attending clinician at the clinic where they are receiving HIV care within each cohort’s legal and ethical framework.

Cohorts with ethics approval and individual patient consent

- Austrian HIV Cohort Study (AHIVCOS): Ethik-Kommission der Medizinischen Universität Wien, Medizinische Universität Graz – Ethikkommission, Ethikkommission der Medizinischen Universität Innsbruck, Ethikkommission des Landes Oberösterreich, Ethikkommission für das Bundesland Salzburg, , Ethikkommission des Landes Kärnten.
- ANRS CO13 HEPAVIH: comite consultative de protection des personnes dans la recherché biomedical, Hopital Tarnier, Paris. ANRS CO3 Aquitaine Biomedical Ethics Committee of the Bordeaux district (’CPP du Sud-Ouest et Outre Mer III’)
- AMACS: Bioethics & Deontology Committee of Athens University Medical School and the National Organization of Medicines
- ICoNA : Comitato Etico San Paolo Azienda Ospedaliera-Polo Universitario San Paolo, Milano
- Modena : Comitato Etico San Paolo Azienda Ospedaliera-Polo Universitario San Paolo, Milano
- PISCIS: Generalitat de Catalunya, department de Salut.
- Swiss HIV Cohort Study (SHCS): Kantonale Ethikkommission, spezialisierte Unterkommission Innere Medizin, Ethikkommission beider Basel, Kantonale Ethikkommission Bern, Comité départemental d’éthique de médecine et médecine communautaire, Commission d’éthique de la recherche clinique, Université de Lausanne, Comitato etico cantonale, Ethikkommission des Kantons St Gallen, Kantonale ethikkomission Zurich, comitatoi etico cantonal, Lugano.
- EuroSIDA study sites (102 actives sites): Before any study related activities are performed Local Ethical Committee approval of the study and procedure for obtaining informed consent from participants is obtained according to local and/or national regulations in all countries participating in the study as well as other national regulatory approvals as applicable. The senior investigator at each clinical site is responsible for obtaining and maintaining this/these approval(s) at all times during the conduct of the study.

Cohorts which, according to national legislation, do not require ethics approvals

- St Pierre
- Cologne-Bonn
- Stichting HIV Monitoring ATHENA cohort (ATHENA)
- VACH
